# What makes us human? Exploring the significance of ricoeur's ethical configuration of personhood between naturalism and phenomenology in health care

**DOI:** 10.1111/nup.12385

**Published:** 2022-03-24

**Authors:** Bengt Kristensson Uggla

**Affiliations:** ^1^ Amos Anderson Professor of Philosophy, Culture, and Management Åbo Akademi University Turku Finland; ^2^ Centre for Person‐Centred Care (GPCC) University of Gothenburg Gothenburg Sweden

**Keywords:** ethics, personalism, person‐centred care, personhood, philosophical anthropology, Ricoeur

## Abstract

The aim of this article is to elaborate on how a distinct concept of the person can be implemented within person‐centred care as an ethical configuration of personhood in the tension between the two predominant cultures of knowledge within health care: naturalism and phenomenology. Starting from Paul Ricoeur's ‘personalism of the first, second, and third person’ and his ‘broken’ ontology, open‐ended, incomplete, and imperfect mediations, placed at the precise juncture where reality is divided up into two separate cultures of knowledge, is identified as crucial for what makes us human. Within this context, Ricoeur's distinct ethical configuration of personhood is based on the homology between the linguistic, practical, narrative, and moral determinations of selfhood—articulated as a hermeneutics of the self, without any methodological break. Person‐centred care is thus recognized as an profound ethical approach to health care based on mediations of ‘horizontal’ (teleological) and ‘vertical’ (deontological) readings of an ethical configuration of personhood by the use of practical wisdom.

## 1 INTRODUCTION

The title I have chosen for my presentation brings to the fore one of the most crucial questions in our time, and a challenging issue within health care as well: what makes us human? In this text, I will mainly draw from the French philosopher Paul Ricoeur (1913–2005) to elaborate on how his concept of the person can be used and implemented within person‐centred care when articulated as an ethical configuration of personhood in the tension between the two predominant cultures of knowledge: naturalism and phenomenology.

The concept person‐centred care invites misunderstandings, and the tremendous success of person‐centred care in Sweden and elsewhere (Britten et al., 2020; Ekman et al., 2011, 2020) has only aggravated the problems associated with maintaining a distinct understanding of the concept. Furthermore, it is difficult to navigate and make clear distinctions in a conceptual landscape in which patient‐centred care, personalized medicine, and person‐centred care often collapse into each other in a very confusing way. Person‐centred care has sometimes also been used to support an almost narcissistic approach when people who consider themselves as the ultimate experts on their own health status claim that a former almighty doctor now is to be replaced by an almighty patient.

Yet, it is exactly in this specific situation, when confusion, misconception, and lack of clear distinctions obscure our mind, that philosophy can make a difference. And when coping with philosophical anthropology and questions like, what is a person? what does it mean to take a person seriously? and what makes us human? Ricoeur seems to be one of the most important philosophers to include in our conversation (Kristensson Uggla, 2010,  2020).

## 2 THE ‘TWO CULTURES’

In a lecture given in 1959, C.P. Snow coined the phrase ‘the two cultures’, referring to a dramatically widening gap between what he identified as *scientists* and *literary intellectuals*: the former represented by forward‐striving positivists (often holding up mathematicians and physics as paradigms), the latter by what he considered opponents of modernization (represented by intellectuals based in the humanities, scholars who did not regard natural scientists as worth engaging in conversation). Snow pointed to a worrying tendency for communication between these two groups to cease entirely, resulting in a disastrous division into what he recognized as two ‘galaxies’ that have lost contact with each other (Snow, 1959). Today, this problematic has worsened and is more difficult and challenging than ever.

The historical background to this aggravated situation is the emergence of what might (anachronistically) be called two parallel ‘scientific’ projects, manifested by the *causal explanations* of the 17th century Scientific Revolution from which the natural sciences emerged, and the revised Aristotelian *teleology* of *understanding* in modern humanities stemming from the tradition of the Medieval university. Nevertheless, it was not until the 19th century that the contours of a dualistic order in which *culture* and *nature*, representing two discrete forms of reality, an ‘inner’ world defined by freedom and teleological purposes and an ‘outer’ world defined by necessity and causality, began to appear as a complex challenge to cope with. Because behind this way of thinking resides an intellectual dichotomy that radically separated soul and body, culture and nature (even if, strictly speaking, only the former had the appearance of ‘culture’—in this asymmetric relationship where the natural sciences increasingly came to dominate the scene) (Kristensson Uggla, 2019).

The cohabitation of these two cultures under a common ‘university umbrella’ has never stopped being uncomfortable. And we live in a time when the clash has become almost painful, between, on the one hand, a naïve, uncritical praise of humanism among politicians and policymakers, not least within the health‐care sector (neglecting the massive critique against the basic assumptions associated with a traditional humanistic approach)—and on the other hand, a theoretical antihumanism not intending to *understand* but *dissolve* human beings, emanating from strong versions of poststructuralist, postmodern, and posthumanist epistemologies, and in particular a predominant empiricist naturalism (neglecting the stark contrast to the private values and often humanistic convictions among scholars themselves) (Kristensson Uggla, 2016).

## 3 NATURALISTIC MEDICINE AND PHENOMENOLOGICAL CARING WITHIN HEALTH CARE

In no other place seems the confrontation—and the experience of disconnect—between the two cultures of knowledge as dramatic as within health care, where a *naturalistic* paradigm of science and research programs in caring inspired by *phenomenology* often develops in parallel systems separated by waterproof bulkheads. How is it possible to overcome this ‘cognitive balkanization’ and articulate what it means to be human in this situation?

Facing these challenges, it is important to remind ourselves, that the anthropological deficit of the naturalistic approach should not be considered a result of decay or some evil thought. The main reason we have ended up in this fragmented and difficult situation is instead connected to the tremendous success of scientific development. So many diseases that recently meant a certain death, can today be easily cured. Thus, medicine appears as something true human—and yet we need more cognitive resources than physiology and biology to understand what it means to be human. Nor can the human condition be identified with an existentialist understanding of freedom as a sheer negating act, and as such entirely reduced to phenomenological experience? Phenomenological investigations are driven by an ambition of ‘humanizing’ knowledge by focusing on some fundamental conditions of knowledge that modern scientific thinking tend to ignore, but in the end of the day, it is yet simply not possible to reduce and trace all knowledge and reality back to a life world constituted by phenomenological experience.

During the pandemic that started in 2020, we also experienced how tremendous successful scientific research based on a naturalistic approach may be when delivering vaccine much faster than anyone thought was possible. Yet, at the same time, we have furthermore been reminded that the challenges associated with mass‐vaccination of an entire population cannot be considered purely medical or biological problems that can be solved by naturalistic methodologies. Learning from the experiences we have done during this period, we should perhaps also be careful to conceptualize the conflict between the two cultures as a confrontation between ‘natural’ sciences and ‘human’ sciences, because these concepts obscure the fact that the ‘human’ sciences alone cannot claim to solely deliver knowledge about what it means to be human. And correspondingly, if person‐centred care should be successful and be able to recognize what it really means to be human, both caring and medicine, phenomenological and naturalistic approaches need to be included (Wallström & Ekman, 2018).

As an alternative to the foundationalist claims of both naturalism and phenomenology—which both forces us to choose between the exclusive claims of two alternative options—Ricoeur has advocated a ‘broken’ ontology using critical hermeneutics ‘bridging the gap’ by *open‐ended*, *incomplete*, and *imperfect mediations* placed at the precise juncture where reality is divided up into two separate cultures. Here, Ricoeur's configuration of historical time has a paradigmatic significance disclosing how true human time appear as a ‘third’ hybrid time established in terms of a series of ‘connections’ by which the phenomenological experience of the *lived* time of the *soul* is inscribed in the *cosmological* (universal) time of the *world* (Ricoeur, 1988). According to the great number of ‘incomplete mediations’ of this ‘broken’ ontology, the unstable fragility of the human condition and the profound vulnerability of everything human should not be considered a problem, but recognized as inevitable parts of what makes us human.

## 4 PHILOSOPHICAL ANTHROPOLOGY IN A POLARIZED LANDSCAPE

Considering the historical background of the two cultures, it may be no surprise that the fundamental structures of philosophical anthropology during the last centuries have as well been extremely polarized, when diverging attempts to establish a *centreing* subject, according to the humanistic tradition, have been opposed by different posthumanist approaches influenced by theoretical frameworks stretching from structuralism to neurobiology but aiming at *decentring* the human being. When considering both these major approaches as impossible to use for a robust understanding of person‐centeredness, where can we find a more balanced understanding of the human condition?

Ricoeur's philosophical journey started in the intellectual soil of French and German existential phenomenology, he then successfully managed to navigate in the anti‐existential (and antihumanistic) cognitive landscape of structuralism, and his thinking finally blossomed in the context poststructuralism. In his defense of human values, he never neglected the massive critique that has been directed against the centred subject, but instead tried to integrate these de‐centreing approaches by developing a third position placed at an equal distance from both Descartes and Nietzsche. Elaborating on what he in the 60's named a *wounded cogito* (Ricoeur, 1970), Ricoeur appropriated the massive critique of the subject from Masters of Suspicion (Marx, Nietzsche, Freud) as well as structuralists and naturalists, together with what can be learned from existential phenomenology and reflexive philosophy, and developed a *hermeneutics of the self* where centreing and decentring approaches to the subject are closely related in terms of a correlation of *intentions*, *causes*, and *coincidences* (Ricoeur, 1992a).

Compared with the polarized positions among thinkers who have contributed to philosophical anthropology during the last century, Ricoeur developed a much more *humble* approach advocating a *fragile* concept of personhood. According to this philosophical anthropology, we need to recognize a person who is *disproportional* with him/herself and constituted by a number of dialectical relationships that makes human identity appear as a *heterogenous synthesis* that can only be recognized by the use of ‘mixed categories’. From where does this approach to the person emanate?

## 5 PERSONALISM? PERSONALISM! THE DEATH OF THE AUTHOR IS THE BIRTH OF THE READER

Already as a young scholar Ricoeur was drawn into the intellectual network around the journal *Esprit*, founded by one of the major representatives of personalism, Emmanuel Mounier (1905–1950), in 1932. The extraordinary importance of Mounier to Ricoeur was made evident by an article he published just after the philosopher had died in 1950 at only 45 years of age, ‘Emmanuel Mounier: A Personalist Philosopher’ (Ricoeur, 1965, pp. 133–161), where he is honouring the memory of the great thinker of personalism: ‘Our friend Emmanuel Mounier will no longer answer our questions […] In a sense, a work attains the truth of its literary existence when its author is dead […] I have not been able to reread Mounier's books as books should be read, as books of a dead person’. (Ricoeur, 1965, p. 133).

It is amazing to note hos Ricoeur in this emotional presentation anticipated elements from his later text hermeneutics when claiming that ‘books should be read, as the books of a dead person’. It might come as a surprise to some, but there is an inherent link between Ricoeur's *philosophy of action* and *the death of the author* in Ricoeur's dialectical understanding of the capable human being. Because to Ricoeur, the ‘death of the author’ (cf. Roland Barthes) never meant to be acknowledged as a theoretical antihumanism declaring the death of the subject in general (Foucault, 1973), in accordance with structuralism and some poststructuralist stand points. The author's original intentions cannot control the words and the interpretation made by readers. But, after the death of the author, nor can the former power of the almighty author as the source of all meaning be transferred to the reader. Text interpretation, according to Ricoeur, should rather be linked to the *dialectic* of *distanciation and appropriation* in the act of reading—and within this framework, the death of the author does not imply the disappearance of *the author as a person*, but rather the death of *the person as author*. When a text is completed the person as author ‘dies’, but he or she does so only to allow the ‘birth’ of the reader—a person capable of performing a cultivated act of reception as well as production in the world in front of the text.

Thus, the fundamental structures of text interpretation have have profound implications for a dialectical understanding of human capacity. And within this dialectical philosophical anthropology ‘the positive and productive function of distanciation at the heart of the historicity of human experience’ (Ricoeur, 2007a, p. 76) is being recognized as an integral part of our understanding of a capable human being. Because the transformation of the author into one among the readers of the text presupposes a self‐understanding which actively includes—and welcomes—a *decentring* of the self, according to the hermeneutical function of distanciation and a recognition that ‘distanciation is the condition of understanding’ (Ricoeur, 2007a, pp. 75 and 88).

In his personalism, Mounier developed a cross‐disciplinary approach aimed at transcending the dichotomies between economic structures and moral values, together with the dualism between body and soul, acting and thinking, homo faber and homo sapiens, generated by the conflict between the ‘two cultures’. By including institutional relationships in his recognition of the person, as a human being incarnated in a community of mutual relationships, Mounier looked for a ‘third position’ intending to reach beyond the dichotomies of individualism and collectivism. Ricoeur takes this project, aiming to transcend the binaries of dialogical and institutional relationships, further into an extended version of personalism, which also contributes to a refiguration of person‐centredness.

## 6 A PERSONALISM OF THE FIRST, SECOND AND THIRD PERSON

Even though he resisted philosophical ‘schools’, it seems possible to recognize Ricoeur's philosophy as an advanced and independent prolongation of Mounier's interrupted personalistic project. Thus, if we want to consider Ricoeur as a personalist thinker, we must distinctly emphasize the contrast to contemporary ‘political’ personalism, together with his affirmation of institutional relationships and the inherent dialectical approach which he disclosed in Mouniers thinking. This has inspired me to name Ricoeur's version of personalism a ‘personalism of the first, second and third person’ (Kristensson Uggla, 2016) as a distinct position which transcends the limitations of dialogue and presents a creative alternative to both humanist and antihumanist approaches, a *wounded cogito* (Ricoeur, 1970), a *homo capax* capable of acting and suffering and imagining ‘oneself as another’.

*Oneself as Another* (Ricoeur, 1992a) is also the title of Ricoeur's most systematic presentation of philosophical anthropology. In this book the recognition of *homo capax*, the capable human being, reaches its highest significance in the conceptualization of the *person* as someone whose actions can be *imputed* to him or her. Here, Ricoeur argues for a philosophy of action where the capable human being is recognized by a profound dialectical approach: the acting human being who is always also recognized as a suffering human being—and this suffering human being is never only considered a victim but also recognized as a person capable of developing actions. Within this dialectical understanding of personhood, *disproportions* stemming from the lack of foundations are present in every aspect of a vulnerable human life, thus indicating the inevitable fragility of everything human.

In his book from 1990 (Ricoeur, 1992a), Ricoeur develops his understanding of *homo capax* as someone who is capable of saying ‘I can’, according to four determinations of human identity and personhood, represented by language (‘I can speak’), action (‘I can act’), narration (‘I can give account for myself’), and ethics (‘I can hold myself responsible for my own actions’). Moreover, each of these four determinations—which together form a *hermeneutics of the self*—has a triadic structure configured by ‘mixed’ and unstable discourses emanating from three different personal pronouns: namely, the first, second and third person—the ‘me’, the ‘you’, and the ‘she/he/it’—including plural equivalents. According to this understanding of personhood, it is fully legitimate to refer to ‘me’ by the variety of personal pronouns: not only ‘me’ speaking in the first person, but also identifying ‘me’ in both second and third person. Moreover, it is legitimate to apply the indirect use of the reflexive pronoun ‘‐self’ in all these cases—‘myself’, ‘yourself’, ‘her/his/itself’—including plural pronominal variations (Ricoeur, 1992a, pp. 1–17). The shift in roles, made possible due to the reflexive character of all personal (and impersonal) pronouns, still referring to one and the same person, reveals a composite communicative structure which relates *self‐understanding* (of a ‘me’ in first person) to *dialogical understanding* (of a ‘you’ of second person), as well as to *objectifying explanations* (in terms of a ‘him/her/it’ in third person). This conceptualization of the person provides us with important resources for developing an understanding of person‐centred care which may transcend the binary of naturalistic and phenomenological approaches. If person‐centred care is considered as an ethical approach, how can this recognition of the human condition based on ‘mixed categories’ be articulated in terms of ethics?

## 7 AN ETHICAL CONFIGURATION OF PERSONHOOD

Ethics is in general considered as something that is externally *added*—a naturalistic approach to human beings and to an existential understanding of personhood. It might seem strange that a philosopher who constantly raised ethical issues never presented a separate book with a systematic presentation of his ethical thinking. Instead of a large comprehensive moral philosophy, we must settle for a *minima moralia*, *une petite éthique*—a ‘little ethics’, presented as an integral part of a wider investigation of personal identity and personhood. Therefore, we need to carefully consider the wider implications of the fact that Ricoeur's ethical aim is not presented in splendid isolation, but as an integral part of a fourfold determination of the self (who speaks?; who acts?; who narrates?; and who is responsible?; later also memory and promise were included).

The homology between the linguistic, practical, narrative and moral determinations of selfhood, described without any methodological break, implies that each of these are integral parts of Ricoeur's hermeneutics of the self. Furthermore, this remarkable continuity is intensified by the fact that their order may be reversed: one may as well start with the ethical determination of the self, continuing with narrative, action and finally language, without losing sight of the dialectic approach to the capacities and incapacities of acting and suffering human beings, possibilities which Ricoeur also elaborated on in other publications (Ricoeur, 1992b).

Ricoeur's first articulation—his ‘horizontal’ reading—of ethics, in terms of an *aim at ‘*the good life*’ with and for others in just institutions* (Ricoeur, 1992a, p. 172), is closely connected to a ‘personalism of the first, second and third person’ (Kristensson Uggla, 2020, pp. 92–99), since it implicates the whole set of possible pronominal variations. Within this context of virtues of *self‐esteem* (first person), *solicitude* (second person) and *justice* (third person), according to the ‘horizontal’ reading the ethical aim, solicitude is not considered as something added onto self‐esteem, as if from the outside, instead solicitude unfolds the implicit dialogical dimension of self‐esteem. Similarly, justice is not recognized as something added onto the other two components: ‘*Equality* […] *is to life in institutions what solicitude is to interpersonal relations*. Solicitude provides to the self another who is a face […] Equality provides to the self another who is an *each*’ (Ricoeur, 2007b, p. 202 [italics added]).

Within this conceptualization of the ethical aim, the connections between *dialogical* and *institutional* relations are characterized by a *discontinuous continuity*: ‘Friendship, however, is not justice, to the extent that the latter governs institutions and the former interpersonal relationships. This is why justice encompasses many citizens, whereas friendship tolerates only a small number of partners’. (Ricoeur, 1992a, p. 184). Here, we may note that Ricoeur's ethical considerations, in contrast to for examples Lévinas and Derrida, includes interpersonal forms of otherness beyond the face‐to‐face encounter of an ‘I’ and a ‘you’ associated with the neutral third perspective of institutions in terms of a variety of juridical, political, technical and financial systems, where the other appears neither as a ‘you’, nor in the realization of the good life in the solicitude of friendship in interpersonal relations, but where the relations to the other challenges us to reflect on how we live together in structures irreducible to interpersonal relations, where the self is identified as ‘each and everyone’. This discloses that person‐centred care has implications far beyond dialogical encounters and also relevant for organizational and societal policies (Öhlén et al., 2017).

## 8 TO BE A CAPABLE HUMAN BEING MEANS RESPONSIBILITY: PRACTICAL WISDOM

In contemporary moral philosophy, Kantian moral philosophy of norms and Aristotelian virtue ethics are often presented as mutually exclusive alternatives. Ricoeur's articulation of ethics in terms of *aiming at the* ‘good life’ *with and for others in just institutions* (Ricoeur, 1992a, p. 172) is grounded in an Aristotelian perspective that combines the *autonomy* and self‐esteem of the self with friendship and *solicitude* for one's neighbour, and a sense of *justice* for ‘each and everyone’. Yet, this ‘horizontal’ (Aristotelian) reading—where human action is birthed through the connections between life, desire, lack and accomplishment—is thereafter combined with a ‘vertical’ (Kantian) reading, motivated by the presence of violence and the wrong that one person inflicts on another, wherein the initial teleological approach, guided by the idea of ‘living well’, intersects with a deontological approach of a second ‘vertical’ axis that qualify human actions in terms of morality. In his ‘vertical’ reading, norms, obligations, and prohibitions form a critical (‘Kantian’) test of the initial (‘horizontal’) aim of living well. This terminology of ‘horizontal’ and ‘vertical’ readings was not present in Ricoeur's original articulation of ethics (Ricoeur, 1992a) but was developed only later (Ricoeur, 2007b; Kristensson Uggla, 2017). It is important to notice that ethics begins as a wish before it becomes a moral imperative. But the ethical aim really needs to be critically evaluated, and therefore must pass through an examination of the norm, duty and interdiction. Thus, the ‘vertical’ dimension of ethics, that is, *morality* (Kant), is for Ricoeur at once *subordinated* and *complementary* to the ‘horizontal’ dimension of ethics, that is, Aristotelian *ethics* (Ricoeur, 1992a, pp. 170–171). Yet, in the end, the deontological ethics cannot provide a solution and tell us how to act in a responsible way. Moral norms can only add—and aggravate—moral dilemmas. But in this way, they also contribute by disclosing the conflicts in the tragic setting of action that can only be handled by practical wisdom, *phronesis*. In opposition to those who expect a presentation of person‐centred care as a standardized manual of rules and principles, this ethical approach to person‐centrered care takes shape as an ethics embedded in practice. Understood in this way, ethics cannot, and should not, ‘solve’ ethical problems and moral dilemmas, but instead cultivate us in the art of making fair decisions in unique situations of great uncertainty. Since conflicts are inevitable—on institutional level as well as on dialogical and individual levels—ethics has to cope with ‘the reflective equilibrium between the ethics of argumentation and considered convictions’ (Ricoeur, 1992a, p. 289) and may in the end only mean *responsibility*. Thus, the ‘course’ of Ricoeur's ‘little ethics’ ends up with nothing else than a person capable of making wise judgements in unpredictable situations characterized by great uncertainty. Thus, if a person‐centred approach intends to transcend the ruinous alternatives of *univocity* and *arbitrariness* in a responsible way, Ricoeur teaches us, that we need to develop well‐informed convictions that can dwell in the moral judgement worthy of the name of *practical wisdom*.

## 9 NARRATIVE MEDIATION AND POETIC LANGUAGE

Today, authoritarian political leaders are sometimes refered to as being ‘person‐centred’. In this situation, the concept person‐centred care invites serious misunderstandings. Because nothing makes people as inhuman as when they are being crooked in themselves. A conceptualization of personhood with a human face needs to include both the capacities and incapacities of a person capable of acting as well as suffering to liberate us from a narcissistic ego by taking us “outside” ourselves. Thus, to understand what it means to be human we need to recognize distanciation and to include conflicts of interpretations from different cultures of knowledge. When dealing with the complexity of this dialectical understanding of the human condition in a distinct manner, we need to include more discourses than scientific concepts and philosophical reflection.

According to the conceptualization of person‐centred care in Ekman et al. (2011), narrative (together with partnership and documentation) plays a crucial role for the understanding of the patient as a person. To travel the journey from the ‘what’ of the patient to the ‘who’ of the person, we need to enter the narrative path. Ricoeur has elaborated on the ontological link between narrative and time: ‘Time becomes human time to the etent that it is organized after the manner of a narrative; narrative, in turn, is meaningful to the extent that it portrays the features of temporal experience’. (Ricoeur, 1984, p. 3).

Poetry is a also powerful tool if we want to understand and communicate what an ethical approach to person‐centred care means in concrete situations, where the profession encounters the patient. Therefore, it seems appropriate to conclude this presentation by refering to the Swedish poet Tomas Tranströmer, using his poetic language for a better understanding of the necessity of establishing a ‘meeting’ that might give us a chance to catch sight of human beings (Tranströmer, 1972, p. 45):

Two truths approach each other. One comes from inside, the other from outside, and where they meet we have a chance to catch sight of ourselves.

In other words, we cannot develop robust self‐understanding and scientific thinking solely on the basis of ‘inner truths’ grounded in our own understanding. We also need ‘outer’ truths in terms of explanations that are based not on ourselves but on distanciated and abstract explanatory models, systems, and theories. In the health‐care sector in particular, the number of ‘outer’ truths seems to have increased exponentially. But to cope with the major challenge to bring about a ‘meeting’ between ‘inner’ and ‘outer’ truths successfully, avoid exclusionary binaries and open up to a fruitful conflict of interpretations, we need to refigure and transform ‘truths’ into ‘interpretations’—that is, hermeneutics.

## 10 CONCLUDING REMARKS

I consider the poetic quote above as the most distinct articulation of a person‐centred approach. In contrast to a traditional humanistic view focused on a centred self, Tranströmer's poetic phrase invites interpretations originating from ‘outside’ according to a de‐centreing of the self. The experience of being de‐centred has multiple meanings: first, the profound experience of receiving life as a ‘outer’ gift; second, the openness to the productive distanciation of different explanations. From the perspective of a ‘broken’ ontology and an ethical configuration of personhood, this kind of ‘meeting’ also indicates the necessity to establish connections linking naturalism and phenomenology together. Human life is instable and fragile, built on heterogeneous syntheses. And when facilitating this kind of ‘meetings’, to be able to cope with the wider conflict of interpretations of a human life where personhood is evitably associated with the capacity to recognize *oneself as another*, practical wisdom is required. To sum up, what actually makes us human seem to be something equivalent with the capacity to establish a meeting‐place between intepretations coming from both ‘inside’ as well as ‘outside’—and thus simulateneously recognize others as human beings.

## CONFLICTS OF INTEREST

The authors declare no conflicts of interest.

## Data Availability

Data sharing is not applicable to this article as no new data were created or analyzed in this study.

## References

[nup12385-bib-0001] Britten, N. , Ekman, I. , Naldemirci, O. , Javinger, M. , Hedman, H. , & Wolf, A. (2020). Learning from Gothenburg model of person centred healthcare. BMJ, 370, m2738. 10.1136/bmj.m2738 32873594

[nup12385-bib-0002] Ekman, I. , Lundberg, M. , Lood, Q. , Swedberg, K. , & Norberg, A. (2020). Personcentrering—en etik i praktiken. In I. Ekman (Ed.), Personcentrering inom hälso‐ och sjukvård (pp. 27–57). Liber.

[nup12385-bib-0003] Ekman, I. , Swedberg, K. , Taft, C. , Lindseth, A. , Norberg, A. , Brink, E. , Carlsson, J. , Dahlin‐Ivanoff, S. , Johansson, I. L. , Kjellgren, K. , Lidén, E. , Öhlén, J. , Olsson, L. E. , Rosén, H. , Rydmark, M. , & Sunnerhagen, K. S. (2011). Person‐centered care‐‐ready for prime time. European Journal of Cardiovascular Nursing: Journal of the Working Group on Cardiovascular Nursing of the European Society of Cardiology, 10(4), 248–251. 10.1016/j.ejcnurse.2011.06.008 21764386

[nup12385-bib-0004] Foucault, M. (1973). The order of things: An archeology of the human sciences. Vintage.

[nup12385-bib-0005] Kristensson Uggla, B. (2010). Ricoeur, hermeneutics, and globalization. Continuum Books.

[nup12385-bib-0006] Kristensson Uggla, B. (2016). Coping with academic schizophrenia: The privileged place of the person when confronting the anthropological deficit of contemporary social imagination: Christian Smith and Paul Ricoeur. In P. Kemp , & N. Hashimoto (Eds.), Eco‐ethica (Vol. 5, pp. 199–218), eds. P. Kemp & N. LIT Verlag.

[nup12385-bib-0007] Kristensson Uggla, B. (2017). A just allotment of memory, Paul Ricoeur on justice and memory. In P. Kemp , & N. Hashimoto (Eds.), Eco‐ethica (Vol. 6, pp. 245–262), eds. P. Kemp & N. H. LIT Verlag.

[nup12385-bib-0008] Kristensson Uggla, B. (2019). En strävan efter sanning: Vetenskapens teori och praktik. Studentlitteratur.

[nup12385-bib-0009] Kristensson Uggla, B. (2020). Personfilosofiska utgångspunkter för personcentrering inom hälso‐ och sjukvård. In I., Ekman (Ed.), Personcentrering inom hälso‐ och sjukvård (pp. 58–105). Liber.

[nup12385-bib-0011] Öhlén, J. , Reimer‐Kirkha, S. , Astle, B. , Håkanson, C. , Lee, J. , Eriksson, M. , & Sawatzky, R. (2017). Person‐centred care dialectics—Inquired in the context of palliative care. Nursing Philosophy, 18e12177, 1–8. 10.1111/nup.12177 28497868

[nup12385-bib-0012] Ricoeur, P. (1965). *History and yruth* (Charles Andrew, trans.). Northwestern University Press.

[nup12385-bib-0013] Ricoeur, P. (1970). *Freud and philosophy: An essay on interpretation* (Denis Savage, trans.). Yale University Press.

[nup12385-bib-0014] Ricoeur, P. (1984). *Time and narrative I*. (Kathleen McLaughlin and David Pellauer, trans.). The Chicago University Press.

[nup12385-bib-0015] Ricoeur, P. (1988). *Time and narrative III*. (Kathleen McLaughlin and David Pellauer, trans.). The Chicago University Press.

[nup12385-bib-0016] Ricoeur, P. (1992a). *Oneself as another* (K. Blamey, Trans.). The University of Chicago Press.

[nup12385-bib-0017] Ricoeur, P. (1992b). Approches de la personne. Lectures 2: La contrée des philosophes (pp. 203–221). Paris.

[nup12385-bib-0018] Ricoeur, P. (2007a). From text to action: Essays in hermeneutics II (K. Blamey, trans.) (p. 134). Northwestern University Press.

[nup12385-bib-0019] Ricoeur, P. (2007b). *Reflections on the just* (D. Pellauer, trans.). The Chicago University Press.

[nup12385-bib-0020] Snow, C. P. (1959). “The two cultures”, The rede lecture. Cambridge University Press.

[nup12385-bib-0021] Tranströmer, T. (1972). “Preludes”, Night vision. Selected and translated from the Swedish by Robert Bly. SelLondon Magazine Editions.

[nup12385-bib-0022] Wallström, S. , & Ekman, I. (2018). Person‐centred care in clinical assessment. European Journal of Cardiovascular Nursing, 17(7), 576–579. 10.1177/1474515118758139 29405743

